# Assessment of nursing home residents in Europe: the Services and Health for Elderly in Long TERm care (SHELTER) study

**DOI:** 10.1186/1472-6963-12-5

**Published:** 2012-01-09

**Authors:** Graziano Onder, Iain Carpenter, Harriet Finne-Soveri, Jacob Gindin, Dinnus Frijters, Jean Claude Henrard, Thorsten Nikolaus, Eva Topinkova, Matteo Tosato, Rosa Liperoti, Francesco Landi, Roberto Bernabei

**Affiliations:** 1 Centro Medicina dell'Invecchiamento, Università Cattolica Sacro Cuore, Rome, Italy; 2 Centre for Health Services Studies, University of Kent, UK; 3 National Institute for Health and Welfare, Helsinki, Finland; 4 Laboratory of Research in Geriatrics and Gerontology, University of Haifa, Israel; 5 EMGO Institute for Health and Care Research, Department of Nursing Home Medicine, VU University Medical Center, Amsterdam, The Netherlands; 6 Research Unit, Health Environment and Ageing, Sainte Pèrine Hospital APHP and Versailles Saint Quentin University Paris, France; 7 AGAPLESION Bethesda Clinic, Competence Center of Geriatrics and Aging Research, University of Ulm, Germany; 8 Department of Geriatrics, 1st Faculty of Medicine, Charles University, Prague, Czech Republic

## Abstract

**Background:**

Aims of the present study are the following: 1. to describe the rationale and methodology of the Services and Health for Elderly in Long TERm care (SHELTER) study, a project funded by the European Union, aimed at implementing the interRAI instrument for Long Term Care Facilities (interRAI LTCF) as a tool to assess and gather uniform information about nursing home (NH) residents across different health systems in European countries; 2. to present the results about the test-retest and inter-rater reliability of the interRAI LTCF instrument translated into the languages of participating countries; 3 to illustrate the characteristics of NH residents at study entry.

**Methods:**

A 12 months prospective cohort study was conducted in 57 NH in 7 EU countries (Czech Republic, England, Finland, France, Germany, Italy, The Netherlands) and 1 non EU country (Israel). Weighted kappa coefficients were used to evaluate the reliability of interRAI LTCF items.

**Results:**

Mean age of 4156 residents entering the study was 83.4 ± 9.4 years, 73% were female. ADL disability and cognitive impairment was observed in 81.3% and 68.0% of residents, respectively. Clinical complexity of residents was confirmed by a high prevalence of behavioral symptoms (27.5% of residents), falls (18.6%), pressure ulcers (10.4%), pain (36.0%) and urinary incontinence (73.5%). Overall, 197 of the 198 the items tested met or exceeded standard cut-offs for acceptable test-retest and inter-rater reliability after translation into the target languages.

**Conclusion:**

The interRAI LTCF appears to be a reliable instrument. It enables the creation of databases that can be used to govern the provision of long-term care across different health systems in Europe, to answer relevant research and policy questions and to compare characteristics of NH residents across countries, languages and cultures.

## Background

About 20% of the population with functional limitations aged 65 and over living in European countries receives long-term care in an institution and about 30% receives formal care at home [1,2]. However, the remaining 50% of individuals receive no formal care and either rely on informal care or receive no care at all. In the next decades, the population with functional limitations is projected to increase by about 120% and the population receiving formal care in institutions will rise by about 130% on average [3].

This rapidly increasing population with chronic disabling conditions will require long term support. It is of crucial importance to understand the health and social care needs of these people and to develop outcome measures for establishing effective and efficient programs and structures. Unlike epidemiological studies on acute hospital care and primary care activity, in long term care there are no sources of comparable data to inform regional or national policies. The issue is seldom the disease diagnosis but rather the functional and social consequences of health conditions [4,5].

In the past decade, the AgeD in the HOme Care (ADHOC) study contributed to the creation of a cross-national database on community care for older people, enabling the possibility of examining quality, outcomes, and trends in home care in Europe [6,7]. Similar evidence on institutional care (nursing home - NH) is lacking in Europe [8,9]. One of the greatest obstacles is the absence of a validated methodology for routinely use in Europe [10]. This need has been clearly identified by the European Union (EU) in its 7^th ^Framework programme [11]. Ideally, such a methodology would support the development of eligibility criteria, ensure standards of services, construct quality indicators, and result in a more efficient resource allocation to grant equal access to services to a rapidly increasing number of individuals.

In the past, a prototype of the current InterRAI instrument for Long Term Care Facilities (interRAI LTCF) - the so called Minimun Data Set - was developed as a comprehensive assessment tool to determine care needs, provide outcome information of NH residents, develop eligibility criteria, support monitoring of services delivery, construct quality measures, and result in a more efficient resource allocation [12]. The new instrument (the interRAI LTCF) is a component of a suite of instruments built around a common set of assessment items relevant to a wide range of health care settings. The common items have identical definitions, observation time frames, and response codes and make these instruments an ideal tool to compare characteristics of older adults across settings [13].

In the US, the systematic use of the interRAI LTCF has led to the creation of a database that has proved of unprecedented value to the study of residents and services characteristics [14]. The possible implementation of such an assessment methodology has never been considered in Europe, other than for research purposes in few countries.

The Services and Health for Elderly in Long TERm care (SHELTER) study addresses the aims of the 7^th ^Framework Programme of the European Union and proposes to validate the interRAI LTCF as an instrument to assess the care needs and provision of care to residents of NH in Europe. The SHELTER study assesses validity and reliability of the InterRAI LTCF instrument when translated in languages of participating EU countries. In addition, the SHELTER study tests the implementation of the instrument on a large scale, leading to the creation of a unique database. These previously unavailable data will support measurements of residents outcomes, development of eligibility criteria, monitoring of services delivery and analysis of quality of care in a European NH setting. This manuscript describes the rationale and methodology of the SHELTER study, highlights the reliability of InterRAI LTCF instrument and illustrates the characteristics of NH residents in the SHELTER database. In addition, it describes a potential application of InterRAI instruments when used on a large scale by illustrating how they enable the comparison of the characteristics of NH residents in the SHELTER with those of older adults in home care in the ADHOC study.

## Methods

The SHELTER study is a project funded by the Seventh Framework Programme of the European Union. The study was performed in 7 EU countries (Czech Republic, England, Finland, France, Germany, Italy, The Netherlands) and 1 non EU country (Israel). Several steps were followed in the study:

### 1. Applicability of the interRAI LTCF to languages of participating countries

The original version of the interRAI LTCF was translated from English version into 7 languages of participating countries (Czech, Dutch, Finnish, French, German, Hebrew, Italian) following several steps: forward translation, reconciliation, back translation, back translation review, cognitive debriefing, review of cognitive debriefing results and proof-reading. This procedure is in line with international recommendations [15,16]. Following this step, in each country, a team of nurses, native speakers of the target languages, were asked to review items included in the translated versions of the interRAI LTCF and to judge whether they reflect the content they are intended to have. The team was composed by eight nurses in most countries, with the only exception of Germany where only five nurses were in the team.

### 2. Training of assessors

Assessors responsible for data collection were trained following a previously validated procedure [17]. In each country, 2-day courses were organised to educate assessors about the concepts of comprehensive geriatric assessment and multidisciplinary teamwork and to train them to the use of interRAI LTCF. In the first part of the course main gerontological and geriatric problems were presented through classes, seminars and conferences. The concept of comprehensive geriatric assessment and multidisciplinary teamwork were introduced and multidimensional assessment tools were presented. In the second part of the course participants learned how to perform the assessment using the interRAI LTCF, including the specific forms and appropriate response codes, and to develop care planning.

Assessors were trained to use a variety of information sources, such as direct observation, interviews with the person under care, family, friends, or formal service providers, and review clinical records, both medical and nursing. The assessors were ordinary clinical staff, external research staff, or a mixture of both. Most assessors were nurses, but other professionals were also used. In line with interRAI's standard approach to coding, they were all instructed to exercise their best clinical judgment in order to record observations based on their evaluation of the most accurate information source.

### 3. Residents enrolment and follow up

**Figure 1:**
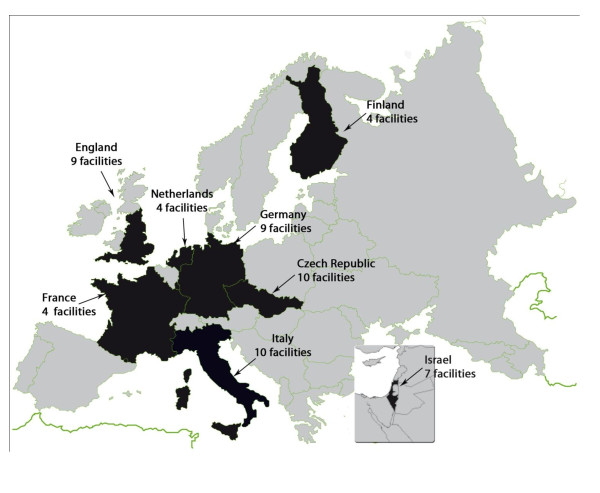
**Geographic distribution of countries participating in the SHELTER study**.

In each country, study partners identified a sample of NH willing to participate to the study. This sample must not be considered randomly selected and is not intended to be representative of all NH in each country. The number of participating facilities in each country is presented in Figure 1.

Older adults residing in participating NH at the beginning of the study and those admitted in the 3 months enrolment period following the initiation of the study were assessed using the interRAI LTCF. In line with previous epidemiological research conducted using interRAI instruments in European populations [6,18], enrolment of a target number of 4000 residents was planned. Participants enrolled in the study were re-assessed at 6 and 12 months if still in the facility. If no longer in the facility, reason (death, hospitalization, discharge at home or in another institution) and date of death or discharge were recorded.

Ethical approval for the study was obtained in all countries according to local regulations. Residents were invited to take part in the study and were free to decline participation. Consent was obtained with assurance of data confidentiality.

### 4. Reliability

A random sample of residents was assessed on two separate occasions within a 2 days period to calculate test-retest reliability and inter-rater reliability. To assess test-retest reliability, the interRAI LTCF was administered to the same residents by the same individual assessors on two different occasions. To assess inter-rater reliability, two independent assessors administered the interRAI LTCF to the same residents independently at different times. Assessors were blinded to others' results and they were not permitted to discuss the case with each other, nor were they permitted to exchange information; however, they were both able to access the persons chart when completing their assessment.

### 5. Analytical Approach

Data from the baseline assessment for the entire study sample are reported. Multi-item summary scales embedded in the interRAI LTCF were used to measure residents characteristics. To evaluate functional status, the seven point MDS Activities of Daily Living (ADL) Hierarchy scale was used [19]. The ADL Hierarchy scale groups activities of daily living according to the stage of the disablement process in which they occur. Early loss ADL's are assigned lower scores than late loss ADL's. The ADL Hierarchy Scale ranges from 0 (no impairment) to 6 (total dependence). ADL disability was categorized as follows: assistance required (ADL Hierarchy Scale score 2 to 4) and dependence (ADL Hierarchy Scale score ≥ 5). The cognitive performance scale (CPS) was used to assess cognitive status [20]. The CPS combines information on memory impairment, level of consciousness, and executive function, with scores ranging from 0 (intact) to 6 (very severe impairment). The CPS has excellent comparability with the Mini Mental State Examination (MMSE) a score ≥ 2 being equivalent to a diagnosis of dementia or a MMSE score of ≤ 19 [21]. Cognitive impairment was categorized as follows: moderate (CPS score 2 to 4) and severe (CPS score ≥ 5). The MDS Depression Rating Scale was used to assess the presence of depressive symptoms and a score ≥ 3 was used to diagnose depression [22]. For all these scales, lower numbers represent less impairment.

The reliability of the categorical interRAI LTCF items was evaluated with weighted kappa coefficients using Fleiss-Cohen weights [23]. According to Landis and Koch, kappa values below 0.40 should be considered poor, between 0.41 to 0.60 should be considered moderate, 0.61 to 0.80 should be considered substantial, and above 0.81 should be considered almost perfect [24]. For continuous variables Pearson's correlation coefficients were presented.

### 6. AdHOC study sample

In order to describe potential application of InterRAI instruments when applied on a large scale characteristics of NH residents in the SHELTER study were compared with those of older adults in home care in the ADHOC study, a project funded by the Fifth Framework Programme of the EU, collecting data on a random sample of 4007 elderly patients admitted to the home care programs in urban areas of 11 European countries, from 2001 to 2003 [6]. The main objective of the AD-HOC study was to identify a model of Home Care for the elderly through the analysis of the structural and organisational characteristics of Home Care Services in different countries of Europe, along with the clinical and functional characteristics of their clients. A trained staff was selected to assess home care patients entering the study by using the InterRAI instrument for Home Care (InterRAI HC). This instrument contains over 350 data elements including socio-demographic variables, numerous clinical items about both physical and cognitive status, as well as all clinical diagnoses. All items are coded using the same assessment approach as for the InterRAI LTCF; namely, assessors used all sources of information and then exercised clinical judgement as to the most appropriate answer based on standardized coding guidelines provided in the instrument's training manual. Most items permit the use of multiple information sources including personal interviews, review of the chart, direct observation of the person, communication with informal caregivers, and use of clinical communication between health care staff. Both InterRAI LTCF and InterRAI HC assessments include a set of common core data elements (e.g. outcomes assessing cognitive or functional status), as well as specialized items unique to that service setting.

In line with previous studies, to compare characteristics of home care patients collected by InterRAI instruments, CPS score and ADL hierarchy scale score are presented [6]. These variables were shown to be strongly related to health outcomes of NH residents [25,26].

## Results

### Sample characteristics

A total number of 4156 residents was enrolled in the study, 500 from Czech Republic, 507 from England, 484 from Finland, 493 from France, 496 from Germany, 580 from Israel, 548 from Italy and 548 from the Netherlands. Table 1 summarizes the principal characteristics of NH residents enrolled in the study. Mean age was above 80 years and women represented approximately 3/4 of the sample. Disability was common, with more than 80% of participating residents requiring assistance or being dependent in ADL and cognitive impairment was present in more than 2/3 of the sample with 30% of residents being classified as severely impaired. Elevated level of "clinical" complexity of residents was confirmed by the high prevalence of urinary incontinence (73.5%), pain (36.0%), depression (32.0%), behavioral symptoms (27.5%), falls (18.6%) and pressure ulcers (10.4%).

**Table 1:** Baseline characteristics of study population.

	Total sample n = 4156 (%)	Czech Republic n = 500 (%)	England n = 507 (%)	Finland n = 484 (%)	France n = 493 (%)	Germany n = 496 (%)	Israel n = 580 (%)	Italy n = 548 (%)	The Netherlands n = 548 (%)
Age, years (mean ± SD)	83.4 ± 9.4	81.3 ± 8.3	84.5 ± 9.5	84.8 ± 8.0	87.3 ± 7.8	84.6 ± 8.3	81.2 ± 11.0	83.5 ± 9.3	81.0 ± 10.4

Female gender	3035 (73)	362 (72.4)	365 (72.0)	362 (74.8)	374 (75.9)	392 (79.0)	412 (71.0)	401 (73.2)	367 (67.0)

ADL disability*									
Assistance required	1723 (41.5)	160 (32.0)	132 (26.0)	293 (60.5)	155 (31.4)	271 (54.6)	195 (33.6)	226 (41.2)	291 (53.1)
Dependent	1653 (39.8)	204 (40.8)	315 (62.1)	129 (26.7)	229 (46.5)	124 (25.0)	293 (50.5)	231 (42.2)	128 (23.4)

Cognitive status†									
Mild/Moderate impairment	1563 (37.6)	213 (42.6)	181 (35.7)	339 (70.0)	141 (28.6)	146 (29.4)	134 (23.1)	152 (27.7)	257 (46.9)
Severe impairment	1265 (30.4)	114 (22.8)	145 (28.6)	82 (16.9)	234 (47.5)	139 (28.0)	273 (47.1)	192 (35.0)	86 (15.7)

Depression‡	1331 (32.0)	145 (29.2)	162 (32.0)	171 (35.9)	171 (34.8)	103 (20.8)	170 (30.5)	193 (36.8)	216 (39.4)

Behavioral symptoms	1142 (27.5)	96 (19.2)	159 (31.4)	224 (46.3)	105 (21.3)	108 (21.8)	160 (27.6)	142 (25.9)	148 (27.0)

Falls	774 (18.6)	131 (26.2)	72 (14.2)	101 (20.9)	95 (19.3)	115 (23.2)	60 (10.3)	75 (13.7)	125 (22.8)

Pressure ulcers	432 (10.4)	79 (15.8)	54 (10.7)	23 (4.8)	58 (11.8)	48 (9.7)	38 (6.6)	73 (13.3)	59 (10.8)

Pain	1496 (36.0)	234 (46.8)	193 (38.8)	222 (46.0)	205 (41.8)	192 (38.8)	92 (15.9)	118 (21.7)	240 (43.8)

Urinary incontinence	3054 (73.5)	354 (69.0)	402 (79.3)	401 (82.9)	362 (73.7)	341 (68.9)	437 (75.3)	417 (76.7)	349 (63.7)

ADL - Activities of Daily Living

* Assistance required is defined by ADL hierarchical scale score 2 to 4, dependent by ADL hierarchical scale score 5 to 6.

† Mild/moderate cognitive impairment is defined by Cognitive Performance Scale Score (CPS) 2 to 4, severe impairment by CPS 5 to 6.

‡ Depression Rating Scale score ≥ 3.

### Test-retest and Inter-rater reliability

Overall, reliability of 201 InterRAI LTCF items was tested. Only 3 items (walking speed, height and weight) were continuous variables. Table 2 presents average weighted kappa values for categorical items in different assessment areas.

**Table 2:** Reliability for InterRAI LTCF items

Assessment Area	Number of items	Average weighted kappa reliability of items in the Area (across country range)	
		
		Test retest reliability (n = 380)	Interrater reliability (n = 404)
Cognition	10	0.88 (0.68 - 0.94)	0.73 (0.52-0.85)

Communication/Hearing	6	0.88 (0.73 - 0.93)	0.79 (0.58-0.89)

Mood/behavior			
- Indicators of depression anxiety and sad mood	14	0.75 (0.51 - 0.95)	0.70 (0.48 - 0.86)
- Behavior symptoms	6	0.82 (0.71 - 0.95)	0.72 (0.46 - 0.91)

Psychosocial well-being	19	0.82 (0.72 - 0.94)	0.72 (0.52 - 0.94)

Physical functioning	18	0.85 (0.62 - 0.95)	0.80 (0.62 - 0.90)

Continence	4	0.87 (0.52 - 1)	0.91 (0.68 - 0.96)

Disease diagnoses	21	0.91 (0.75 - 0.96)	0.76 (0.62 - 0.89)

Health conditions			
- Falls	2	0.82 (0.66 - 0.98)	0.76 (0.49 - 1)
- Problems/conditions	22	0.78 (0.66 - 0.90)	0.66 (0.46 - 0.88)
- Pain	5	0.81 (0.54 - 0.96)	0.70 (0.55 - 0.88)
- Instability of conditions	3	0.86 (0.64 - 0.95)	0.65 (0.41 - 0.87)
- Self-reported health	1	0.82 (0.72 - 0.91)	0.70 (0.56 - 0.80)

Oral/Nutritional Status	12	0.81 (0.65 - 0.93)	0.68 (0.52 - 0.80)

Skin conditions	7	0.79 (0.63 - 0.99)	0.74 (0.44 - 0.88)

Medication	1	0.92 (0.75 - 0.98)	0.85 (0.70 - 0.95)

Treatments and procedures			
- Prevention	8	0.87 (0.72 - 0.98)	0.70 (0.42 - 0.91)
- Treatments	14	0.88 (0.69 - 0.97)	0.83 (0.66 - 0.92)
- Restrictive devices	3	0.86 (0.65 - 0.97)	0.79 (0.69 - 0.89)

Discharge potential and overall status	4	0.91 (0.79 - 0.98)	0.77 (0.63 - 0.85)

Activity Pursuit	18	0.80 (0.54 - 0.95)	0.64 (0.44 - 0.95)

Test-retest reliability was evaluated in 380 residents by 48 assessors (nurses: 42; MD: 5, psychologist: 1) in 17 facilities. This sample was composed of 52 dual assessments from Italy, 50 from Czech Republic, Germany and Israel, 48 from England and France, 43 from Finland and 39 from the Netherlands. As shown in table 2 test retest reliability of InterRAI LTCF items in each of the assessment areas ranged from 0.75 to 0.92. Average weighted kappa for the 198 categorical items was 0.83. None of the single items in the InterRAI LTCF presented with a kappa value below 0.40. Pearson's correlation coefficients for items based on continuous variables were 0.96 for walking speed, 0.92 for height and 0.96 for weight.

Inter-rater reliability was evaluated in 404 residents by 76 assessors (nurses: 68; MD: 4; psychologists: 4) in 23 facilities. This sample was composed of 60 dual assessments from Israel, 59 from Italy, 50 from England, 49 from France and Czech Republic, 48 from Germany and Finland and 41 from the Netherlands. As shown in Table 2 inter-rater reliability of items in the assessment areas ranged from 0.64 to 0.91. Average weighted kappa for the 198 categorical items was 0.74. Only one single item had a kappa value below 0.40 ('Fluid output exceeds input' included in the Oral/Nutritional Status assessment area). Pearson's correlation coefficients for items based on continuous variables were 0.88 for walking speed, 0.99 for height and 0.89 for weight.

### Comparison of SHELTER and ADHOC samples

**Figure 2:**
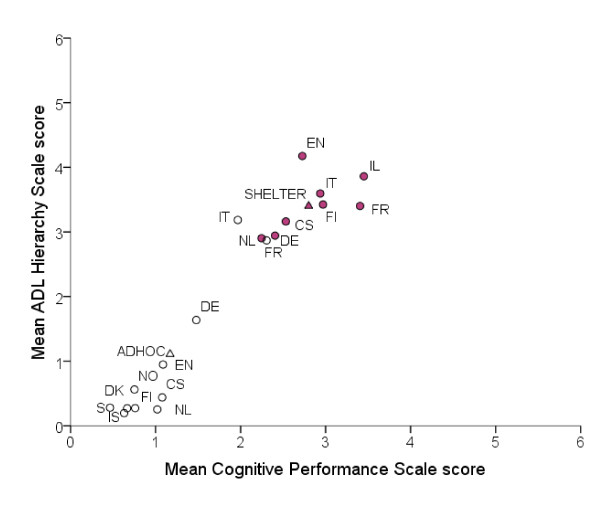
**Relationship between mean Cognitive Performance Scale score and mean ADL hierarchy scale score by country in the SHELTER (full dots) and ADHOC (empty dots) samples**. CS = Czech Republic, DE = Germany, DK = Denmark, EN = England, FI = Finland, FR = France, IL = Israel, IT = Italy, IS = Iceland, NL = The Netherlands, NO = Norway, S = Sweden. Triangles represent mean values in the SHELTER (full triangle) and ADHOC (empty triangle) samples.

Figure 2 compares cognitive and physical functional characteristics (as measured by the CPS scale score and the ADL hierarchy scale score, respectively) of NH residents participating in the SHELTER study and home care recipients participating in the ADHOC study. Both these samples were assessed with InterRAI instruments.

NH residents in the SHELTER project showed higher cognitive and physical impairment than home care recipients in the ADHOC study. In all the countries participating in both studies, a higher level of cognitive and physical impairment was seen in NH residents compared with home care recipients. Interestingly, home care recipients in Italy and France had levels of cognitive and physical impairment similar to the NH residents in the Netherlands, Czech Republic and Germany.

## Discussion

The SHELTER study shows that the interRAI LTCF 1. is a reliable instrument which can be used to assess characteristics of NH residents; 2. can be used to compare characteristics of NH residents across Europe; 3. may contribute to the collection of information and to the creation of large data sets.

InterRAI LTCF was designed to assist clinicians in assessing the care needs of NH residents and providing a comprehensive view of the needs and strengths of the NH population, a view that is essential to the development of appropriate plans of care and allocation of resources [13]. It shares with other interRAI instruments a common set of assessment items that are considered to be important in all care settings [27]. This methodology may improve continuity of care and provide the essential basis for the most effective use of information technology [28]. The use of these standardized instruments gives an opportunity to physicians, therapists, nurses, families, advocates, administrators, and public payers to track changes in an older adult's status across settings and over time. Completion of a full assessment based on InterRAI instruments may take between 20 and 90 minutes depending on setting and assessor experience with the use of the instrument: in particular it has been estimated that between 60 and 90 minutes are necessary to complete an initial InterRAI LTCF assessment [17,29]. Each of these instruments has been developed to be rooted in care planning through a triggering system that enables the identification of the person's problems, but also provides information for monitoring quality of care, case-mix systems, and eligibility screeners.

### Reliability

Virtually all of the items tested met or exceeded standard cut-offs for acceptable test-retest and inter-rater reliability after translation into target languages and a substantial proportion of items showed excellent reliability. These results demonstrate that when implemented by appropriately trained and motivated staff the interRAI LTCF can provide high quality assessment as part of normal clinical practice. Similar reliability levels were found in previous studies performed using interRAI instruments in different settings, suggesting that these instruments, when appropriately translated and validated can be applied in different settings and geographic regions [30-33].

In a recent study Hirdes and coll. tested inter-rater reliability of InterRAI LTCF in 246 nursing home residents, finding kappa values ranging from 0.63 and 0.96. Interestingly, in line with the present study, the items with the best mean kappa values were continence (k = 0.90), physical functioning (k = 0.87) and medications (0.91) [30]. The reliability of InterRAI instruments was also excellent when tested in other settings, including home care (average weighted kappa of items in the instrument = 0.74) [31], acute care hospitals (k = 0.82) [32] palliative care (k = 0.80) [33] and postacute care (k = 0.73) [30].

To assess inter-rater reliability, in line with previous publications [30-33], we have chosen to have independent assessors rating different patients in different facilities and countries, rather than having all study assessors in different countries evaluating the same patient (i.e. by the use of video recordings or clinical vignettes). This approach was chosen in order to minimize potential problems related to the creation of standardized assessment material in different study languages (video recordings or clinical vignettes).

### Comparison

The use of a standardized methodology enables comparisons of older adults across regions and settings. As shown in the manuscript (Figure 2), data of home care recipients collected by interRAI community care assessment instrument (interRAI HC) in the ADHOC study can be directly compared with those of NH residents in the SHELTER study. The SHELTER sample is not meant to be nationally representative and therefore residents characteristics can not be generalized to all NH residents in European countries. However, the data presented indicate the potential of interRAI instruments when applied on a large scale.

The International Classification of Function, Disability and Health (ICF) is intended to provide comparisons of physical, mental and social function, the factors that are the expression of impact of chronic disease [34]. However to date, the absence of reliable assessment systems that can deliver ICF classification has meant that there has been no means of generating population Ievel ICF data. SHELTER has shown that the interRAI LTCF is a reliable tool in EU NH. ADHOC demonstrated the same for EU recipients of community care. Future research should explore the extent to which ICF classifications can be created from these assessment systems to enable the generation of internationally comparable epidemiological data in our aging populations characterized by the increasing prevalence of chronic diseases [35].

### Datasets

Systematic implementation of interRAI LTCF can contribute to the collection of information and to the creation of large data sets. InterRAI instrument based data sets provide a resource to support the analysis of crucial issues pertinent to the clinical care of very old, complex, chronically ill patients living in different settings, especially in NH and in the community. As these individuals are generally excluded from clinical trials, physicians and the health personnel have to rely their best judgment when making decisions. Linking reliable and valid outcome measures constructed with the interRAI LTCF items to the interventions received--pharmacological and nonpharmacological-- opens a new field of outcome oriented geriatric research relevant to scientists, clinicians, and administrators [36].

The data sets have become an important resource for filling knowledge gaps in these understudied populations. Older adults with multiple chronic conditions are still systematically excluded from clinical trials that inform the evidence base for medicine. Nowadays, these clinically relevant data sets are commonly used to conduct observational studies which, when appropriately performed, have proved as valid and useful as randomized clinical studies [37-40]. The creation of a SHELTER database containing data from the interRAI LTCF assessment and information on medications may enabled the exploration of a breadth of clinical questions regarding syndromes such as falls, pain, prevalent medical conditions and geriatric syndromes. Data in the SHELTER database may be useful to identify prognostic factors, and assess patients outcomes and quality indicators Results based these analyses may provide guidance to develop programs to improve quality of care in NH.

## Conclusions

In conclusion, the SHELTER study validates the interRAI LTCF as a methodology that can be used not only to assist clinicians in performing a comprehensive assessment of NH patients, but also to compare characteristics of older adults across countries and to collect information and create databases that can be used to answer relevant questions in the field of long term care. This new methodology can be applied on a large scale to analyze the provision of long-term care across European health systems.

## Competing interests

IC, HFS, JG, DF, JCH, ET and RB are members of InterRAI.

## Authors' contributions

GO, RB: conception and design, analysis and interpretation of data, preparation of the manuscript; IC, HFS, JG, DF, JCH, TN, ET: acquisition and interpretation of data, revision of the manuscript; MT, RL, FL: concept and design, acquisition and interpretation of data, revision of the manuscript. The final version of the manuscript was revised and approved by all authors.

## Contributors

*Czech Republic*: Fialova D, Madlova P, Vlachova M, Ramadhani Chamradova MK. *Germany*: Denkinger M, Lukas A, Rissman U. *England*: Huang Y. *Finland*: Alanen HM, Björkman M, Hosia-Randell H, Elomaa-Sulkava U, Muurinen S, Noro A, Pitkälä K. *France*: Pasek J, Dequesne M, Armand MT, Berrabah MM, Logé O, Le Dastumer MD, Dugny G, Champvert P. *Israel*: Epstein S, Levi S, Ben-Israel S. *Italy*: Danese P, Antocicco M, Fusco D, Menculini G, Borroni G. *The Netherlands*: van der Roest H

## Pre-publication history

The pre-publication history for this paper can be accessed here:

http://www.biomedcentral.com/1472-6963/12/5/prepub
